# The Comparative Protective Effects of Ganoderma Spores Lipid and Fish Oil on N-Methyl-N-Nitrosourea-Induced Photoreceptor Cell Lesion in Rats

**DOI:** 10.1155/2011/903261

**Published:** 2011-04-17

**Authors:** Yang Gao, Xin-Guo Deng, Na Li, Guang-Wei Luo, Peter C. K. Chung

**Affiliations:** ^1^State Key Laboratory of Ophthalmology, Zhongshan Ophthalmic Center, Sun Yat-Sen University, 54 Xianlie Road South, Guangzhou 510060, China; ^2^Shanghai Ninth People's Hospital, Shanghai Jiao Tong University School of Medicine, 639 Zhizaoju Road, Shanghai 200011, China

## Abstract

*Purpose*. To compare Ganoderma spores lipid (GSL) and fish oil (FO) in inhibiting retinal photoreceptor cell lesions induced by N-methyl-N-nitrosourea (MNU) in rats. *Methods*. 120 rats were untreated (normal control, NC group) or treated with a single intraperitoneal injection of 40 mg/kg MNU (MNU group) then treated with GSL (GSL group) or FO (FO group). Eyes were obtained at 1, 3, 5, 7, and 10 days. *Results*. Light microscopy assay demonstrated that GSL and FO alleviated rat retinal photoreceptor cell damage (GSL and FO versus MNU group *P* < .001) similarly (GSL versus FO group *P* = .980). Electron microscopy confirmed that GSL and FO reversed damage to photoreceptor segments and photoreceptor cell nuclei. GSL-treated rats showed significantly elevated a-wave and b-wave amplitudes over MNU group (*P* < .05) but less than NC group (*P* < .05) and not significantly different from FO group (*P* > .05). *Conclusion*. GSL, like FO, alleviates rat retinal photoreceptor cell damage induced by MNU.

## 1. Introduction

Retinitis pigmentosa (RP) is a heterologous group of inherited human disorders causing primary retinal degeneration and functional blindness. The main feature of RP is progressive damage to the retinal photoreceptor and pigmentary epithelium [1]. The incidence of this disease is about 1/4000, and almost 1.5 million are affected persons worldwide [2]. While effective medicines to treat RP are in various stages of development, none are generally available.

A number of studies published in recent years have examined the potential protective effects of fish oil (FO) supplementation in ocular disease. FO is an excellent source of n-3 polyunsaturated fatty acid (PUFA). Both statistically and clinically, significant improvement in visual acuity or stability was observed in patients with intermediate age-related macular degeneration (AMD) treated with a nutritional supplement rich in n-3 PUFA [3]. FO has a therapeutic effect on the degeneration of the retinal photoreceptor cell induced by light in an animal model [4]. Although it has a variety of health benefits, FO is not always well tolerated. While the most common side effects of taking FO supplements are gastrointestinal upset and fishy aftertaste, and it can also cause excessive bleeding or heavy metal poisoning [5].

Ganoderma lucidum (G. lucidum Reishi) is a medicinal mushroom used for centuries in East Asian countries to promote health and longevity. However, only in the last ten years have Ganoderma lucidum spores (5–12 *μ*m) been available for the same medical purpose as the whole mushroom. More recently, the lipid stored between the inner and outer walls of spores, Ganoderma spores lipid (GSL) has been successfully extracted from sporoderm-broken germinating spores [6, 7]. Interestingly, GSL concentrates all the bioactive components of the spores [7, 8], whose bioactive may be much higher than those of the fruiting body of Ganoderma lucidum [6]. Recent studies have demonstrated the neuroprotective effect of Ganoderma lucidum to reduce oxidative stress in vitro [9], to induce neuronal differentiation [10], and to prevent the harmful effects of the exterminating toxin A_*β*_ in Alzheimer's disease in cultured rat neurons [11]. However, other effects of GSL on the nervous system have not been explored, and even less is known about its effect on retina degenerative diseases.

In our previous study, we found that GSL blocked N-methyl-N-nitrosourea- (MNU-) induced photoreceptor cell apoptosis in rats via regulating Bax, Bcl-xl, and caspase-3 [12]. Importantly, GSL contains a great number of PUFAs [7], just like FO. But the majority composition in GSL is oleic acid (OA) while in FO is docosahexaenoic acid (DHA). Both GSL and FO exhibit the protective effects on photoreceptor cells. So, the purpose of the present research was to investigate the protective effect of GSL relative to FO in reversing MNU-induced photoreceptor cell damage in rats.

MNU-induced photoreceptor cell damage in rats provides a useful model for understanding RP and developing and evaluating therapeutic interventions [13]. In the present study, a single intraperitoneal dose of 40 mg/kg MNU was used to induce retina damage and photoreceptor cell loss. Via electrophysiology and morphology analysis, our findings show that GSL has the same effect as FO in alleviating the extent of rat retinal photoreceptor cell damage induced by MNU.

## 2. Materials and Methods

### 2.1. Animals and Procedures

One hundred and twenty 50-day-old female Sprague-Dawley rats were obtained from the animal facility at the Sun Yat-sen University, Guangzhou, China. The experiments were conducted in compliance with the ARVO Statement for the Use of Animals in Ophthalmic and Vision Research, and the study was approved by the local Medical Ethics Committee. Rats were reared under standard laboratory conditions (22  ±  2°C, 60%  ±  10% relative humidity and a 12-hr light-dark cycle) and had free access to food and water throughout the experiment.

**Figure 1:**
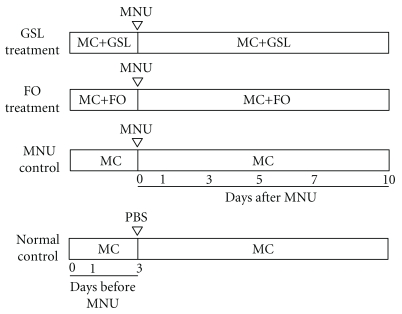
Schematic representation of animal groups and experimental protocol. Rats were randomly divided into the normal control (NC) group (NC group), the untreated N-methyl-N-nitrosourea (MNU) model control group (MNU group), the Ganoderma spores lipid (GSL) treatment group (GSL group), and the fish oil (FO) treatment group (FO group). We used 0.5% hydroxypropyl methyl cellulose (MC) as the excipient. ▿ Intraperitoneal injection was of 40 mg/kg MNU or 4 mL/kg PBS. Six rats were sacrificed at each time point after MNU or PBS injection.

The rats were randomly divided into four groups (Figure 1), including the normal control group (NC group, *n* = 30), the untreated MNU model control group (MNU group, *n* = 30), the GSL treatment group (GSL group, *n* = 30), and the FO treatment group (FO group, *n* = 30). According to our previous work [12], the dose of GSL (BXLC 070116, specific gravity 0.9173, Holistol International Ltd., Hong Kong, China) used was 2 mg/kg body weight by intragastric administration once daily. GSL was mixed in 1 mL 0.5% hydroxypropyl methyl cellulose (MC) used as the excipient. Intragastric administration of GSL was performed each day for the three days before the animal model was established. One hour after the administration of GSL on day three, a single intraperitoneal injection of 40 mg/kg MNU (Sigma, St. Louis, Mo) was given to establish the animal model. MNU, kept at −20°C in the dark, was dissolved in PBS to a final concentration of 10 mg/mL immediately before use. GSL was consecutively given to animals daily for ten days after MNU injection. In the NC group, rats received intragastric administration of excipient (1 mL once daily) instead of GSL and intraperitoneal injection of 4 ml/kg PBS instead of MNU compared with the GSL group. In the MNU group, the excipient (1 mL once daily) was intragastric administrated instead of GSL. In the FO group, rats received intragastric administration of FO (Incromega DHA 500TG SR, Croda, UK, 2 mg/kg once daily) instead of GSL. One hour after administration of GSL, or FO, or MC on days 1, 3, 5, 7, and 10 post-MNU or PBS injection in each group, the left eye of six randomly selected animals was chosen for electroretinogram (ERG) analysis. The amplitudes of a- and b-waves were analyzed. After ERG analysis, animals were sacrificed, and right eyes were prepared for analysis by light microscopy and electromicroscopy. 

### 2.2. Electroretinogram (ERG)

As described previously [12], rats were weighed and dark-adapted for 2 hr before ERG analysis. ERG wavelets were recorded four times, and the average amplitudes of a- and b-waves were calculated. The amplitude of the a-wave was measured as the distance from the baseline to the bottom of the a-wave, while the amplitude of the b-wave was measured as the distance from the bottom of the a-wave to the peak of the b-wave. ERGs were monitored by electrophysiological recorder (Neuropack *α*, Nihon Kohden Corp., Tokyo, Japan) at different times after MNU injection in each group.

### 2.3. Light Microscopy Assay

The right eyes were prefixed with 4% paraformaldehyde in PBS at room temperature for 30 min. The eyeballs were sagittally cut into two half-balls through the optic nerve head. One half was reserved for electromicroscopy assay (described below). The other half was fixed in methacarn solution for 24 hr [14], and the lens was extirpated. Next, samples from this half were incubated in 50% ethanol for 1 hr, dehydrated, and embedded routinely, and sagittal sections (4 mm thick) were cut near the optic nerve head. The slices were dewaxed, stained with haematoxylin for 10 min, restained with eosin for 5 sec and sealed with neutral gum. The extent of damage to the retinal photoreceptor cell was observed under optical microscope (HB-10104A, Nikon Corp., Tokyo, Japan) and photographed. The extent of retina damage to the outer nuclear layer (ONL) was evaluated under the microscope and was graded on a pathological score scale of 0–5 using criteria based on the extent of ONL damage as described in Table 1.

### 2.4. Electromicroscopy Assay

The half of the right eye reserved for electromicroscopy assay was kept in 2.5% glutaraldehyde overnight. The samples were postfixed in 2% osmium solution 20 min, then dehydrated and embedded in epon, and polymerased at 60°C. Thin sections (50 nm thick) stained with uranyl acetate and lead citrate were examined using a Tecnai Spirit G^2^ electron microscope (FEI, Hillsboro, Ore).

### 2.5. Statistical Analysis

The data were presented as the mean ± standard deviation (SD). Statistical analyses were performed using one-way ANOVA followed by Bonferroni-test or Dunnett T3-test employing SPSS version 11.0 (SPSS, Inc., Chicago, Ill). The significance level was set at *P* < .05.

## 3. Results

### 3.1. GSL Shows the Same Protective Effect with FO on Retina Function Detected by Electroretinogram

**Figure 2:**
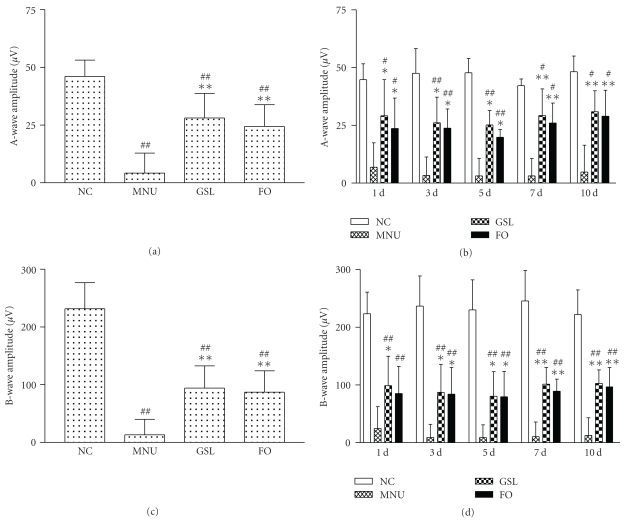
The effect of Ganoderma spores lipid (GSL) and fish oil (FO) on the decrease in a- and b-wave amplitude in N-methyl-N-nitrosourea- (MNU-) induced retina damage in rats. (a) A-wave amplitudes of normal control (NC), MNU, GSL, and FO groups; (b) a-wave amplitudes at each time point for NC, MNU, GSL, and FO groups; (c) b-wave amplitudes of NC, MNU, GSL, and FO groups; (d) b-wave amplitudes at each time point for NC, MNU, GSL, and FO groups. After MNU, all eyes showed reductions in both a- and b-wave amplitude compared with the NC group. In rats receiving GSL, both amplitudes decreased significantly less than those of rats in the MNU group, but no significant change was found between the GSL group and the FO group. These data suggest that GSL, similar to FO, has a protective effect on MNU-damaged retinal ERG. *P < .05 versus MNU, **P < .01 versus MNU, ^#^ *P* < .05 versus NC, ^##^ *P* < .01 versus NC.

In the MNU group, both a- and b-wave amplitudes were decreased significantly at each time point compared with the NC group (all *P* < .01, Figures 2(b) and 2(d)). In GSL-treated rats, both a- and b-wave amplitudes were elevated significantly at each time point compared with the MNU group (*P* < .05, Figures 2(b) and 2(d)) but still lower than the NC group (*P* < .05 or  .01, Figures 2(b) and 2(d)). Meanwhile, in FO-treated rats, both a- and b-wave amplitudes were basically the same as in the GSL group (*P* > .05). In brief, GSL-treated eyes exhibited more moderate loss in both a- and b-wave amplitudes compared with MNU control (*P* < .01, Figures 2(a) and 2(c)), and the loss was statistically significantly similar for the GSL group and FO group (*P* > .05, Figures 2(a) and 2(c)).

### 3.2. GSL, Similar to FO, Inhibits Retina Damage Induced by MNU Observed under Light Microscopy

**Figure 3:**
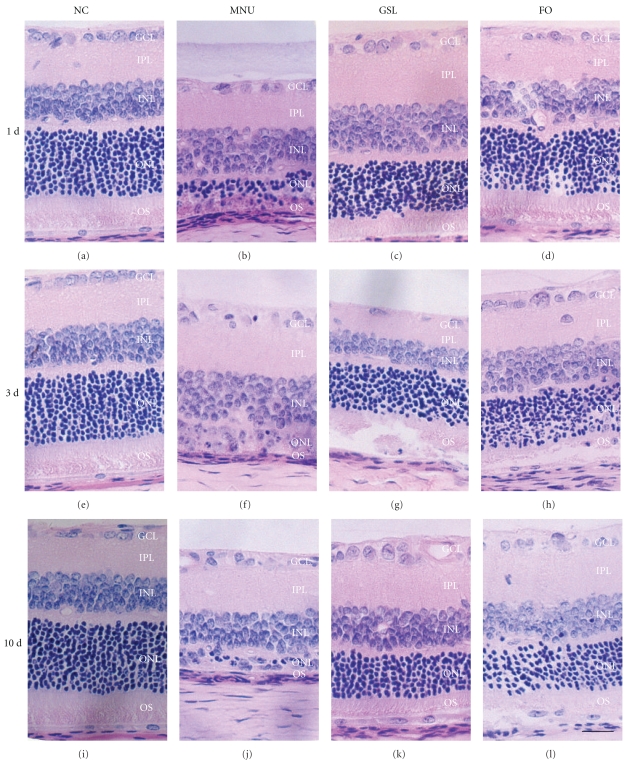
The H-E staining photographs of rat retina treated with Ganoderma spores lipid (GSL) and fish oil (FO). (a, e, i) Normal control (NC) group on days 1, 3, and 10, respectively; (b, f, j) N-methyl-N-nitrosourea (MNU) treated group on days 1, 3, and 10, respectively; (c, g, k) GSL group on days 1, 3, and 10, respectively; (d, h, l) FO group on days 1, 3, and 10, respectively. A single systemic administration of MNU evoked progressive retinal lesions. GSL and FO treatment caused inhibition of retinal lesions induced by MNU. GCL, ganglion cell layer; IPL, inner plexiform layer; INL, inner nuclear layer; ONL, outer nuclear layer; OS, outer segment. Scale bar, 40 *μ*m.

A single systemic administration of MNU evoked progressive retinal lesions in all MNU treated rats, while NC rats retained normal retinal appearance. As shown in Figure 3, compared with retinas from the NC group (Figures 3(a), 3(e), and 3(i)) retinas from MNU-treated animals (Figures 3(b), 3(f), and 3(j)) showed an apparent reduction in ONL thickness and distorted nuclear orientation. GSL treatment (Figures 3(c), 3(g), and 3(k)) significantly inhibited the retinal lesions induced by MNU, as did FO treatment (Figures 3(d), 3(h), and 3(l)).

**Figure 4:**
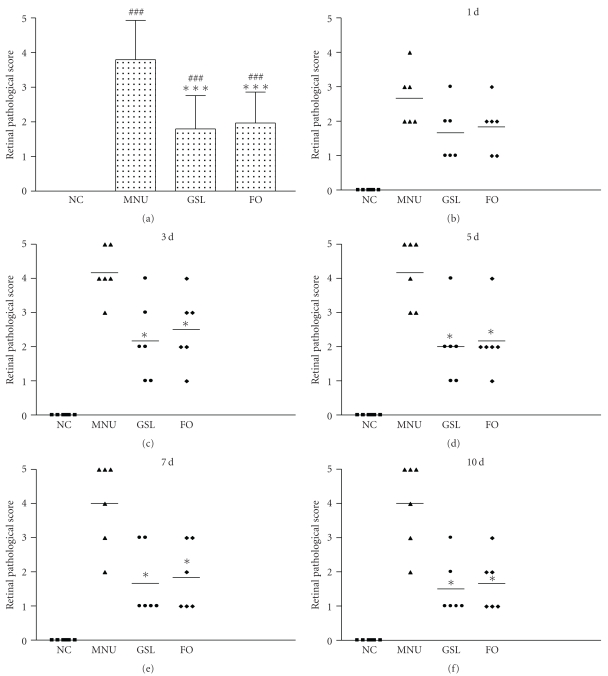
The relative protection effects of Ganoderma spores lipid (GSL) and fish oil (FO) as reflected by the pathological score of rat retina. (a) Retina pathological scores of rats in the normal control (NC), N-methyl-N-nitrosourea (MNU), GSL, and FO group; (b) scores on day 1; (c) scores on day 3; (d) scores on day 5; (e) scores on day 7; (f) scores on day 10. Retina pathological score was evaluated by histopathology (H-E staining) on days 1, 3, 5, 7, and 10 after MNU injection. Each point represents the score of one rat (right eye). Scores from GSL- and FO-treated eyes were significantly lower than those from MNU-treated eyes on days 3, 5, 7, and 10 after MNU (*P* < .05). There was no significant difference in scores of GSL- and FO-treated eyes (*P* > .05). The horizontal bar denotes the average of each group. *P < .05 versus MNU, ***P < .001 versus MNU, ^###^ *P* < .001 versus NC.

Retina pathological score was evaluated by histopathology (H-E staining) and scored (Figure 4). As shown in Figure 4(a), MNU injection caused significantly higher scores than in the NC group (*P* < .001). After treatment with GSL, rats developed less retinal pathology than those in the MNU group (*P* < .001) but still higher than in the NC group (*P* < .001); disease scores of GSL-treated rats were similar to those of FO treated rats (*P* = .98 versus GSL group, *P* < .001 versus MNU group). Meanwhile, retina pathological score was evaluated on days 1, 3, 5, 7, and 10 after MNU injection (Figures 4(b)–4(f)). Scores from GSL- and FO-treated eyes were significantly lower than those of MNU-treated eyes at days 3, 5, 7, and 10 (*P* < .05). GSL- and FO-treated eyes did not differ significantly in scores (*P* > .05). 

### 3.3. GSL, Similar to FO, Inhibits Retina Damage Induced by MNU Observed under Electron Microscope

**Figure 5:**
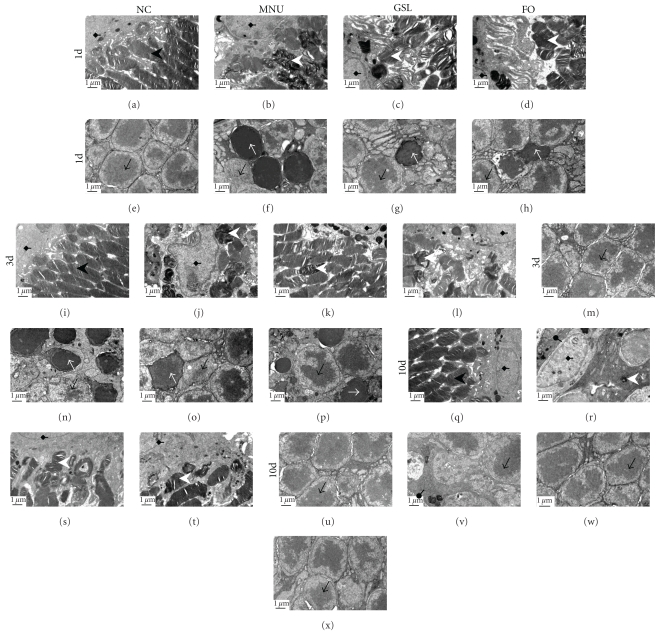
The protection effect of Ganoderma spores lipid (GSL) and fish oil (FO) as shown by electron microscopy on days 1 (a–f), 3 (i–p), and 10 (q–x) for normal control (NC) group (first column) and eyes treated with N-methyl-N-nitrosourea (MNU, second column). Retina in the GSL group (third column) showed milder damage in photoreceptor segments and less nuclear apoptosis than those in MNU group. Retina in the FO group (fourth column) had the same characteristics with those in GLS group. Diamond arrow: pigment epithelium nucleus. Black arrowhead: normal photoreceptor segments. White arrowhead: damaged photoreceptor segments. Black arrow: normal nucleus in the outer nuclear layer. White arrow: apoptosis nucleus in the outer nuclear layer. Round arrow: Bruch's membrane. Scale bar, 1 *μ*m. Magnification: 9700x. These suggest that both GSL and FO treatment can inhibit retinal lesions induced by MNU.

At all detected time points, photoreceptor segments were intact (Figures 5(a), 5(i), and 5(q)), and photoreceptor cell nuclei were normal in appearance (Figures 5(e), 5(m), and 5(u)) in the NC group. On day 1 after MNU, in the MNU group, photoreceptor segments were severely damaged and being phagocyted (Figure 5(b)), and fragmented photoreceptor cell nuclei were seen (Figure 5(f)). In the GSL group, photoreceptor segments were mainly intact (Figure 5(c)), and photoreceptor cell nuclei were less fragmented than in the MNU group (Figure 5(g)). Similarly, in the FO group, photoreceptor segments were slightly damaged (Figure 5(d)) and photoreceptor cell nuclei were less fragmented than in the MNU group (Figure 5(h)). On day 3 after MNU, photoreceptor segments were completely damaged or lost in the MNU group (Figure 5(j)), and plenty of fragmented photoreceptor cell nuclei were seen (Figure 5(n)). In the GSL group, photoreceptor segments were largely damaged and lost (Figure 5(o)), and some photoreceptor cells were damaged (Figure 5(k)); slightly more damage could be seen in the FO group (Figures 5(l) and 5(p)) than in the GSL group. On day 10 after MNU, photoreceptor segments were completely lost in the MNU group (Figure 5(r)), and the most of the photoreceptor cells were lost (Figure 5(v)). In GSL- (Figures 5(s) and 5(w)) and FO-treated rats (Figures 5(t) and 5(x)), photoreceptor segments were disorganized, and many photoreceptor cells were lost, but no fragmented photoreceptor nuclei could be seen.

## 4. Discussion

The mechanism underlining MNU-induced retinal outer nuclear layer damage is that MNU specifically targets the retinal photoreceptor cell, restricting the inward turning of nuclear DNA, resulting in apoptosis of the photoreceptor cell [15, 16]. A single systemic administration of the alkylating agent MNU in rats leads to apoptosis and photoreceptor cell loss within approximately seven days [15]. We, therefore, observed the performances of rats for 10 days after MNU.

In the dark-adapted flash ERG, the a-wave primarily represents the mixed function of cones and rods, while b-wave mainly reflects light-induced depolarization of bipolar cells and the activity of other cells, including Müller cells [17]. Firstly, we illustrated the protective effects of GSL and FO on retina by ERG assay. Our data showed that after daily administration of GSL, rats exhibited more moderate loss in both a- and b-wave amplitudes than rats that received only MNU injection. The protective effect of GSL on the retina was not significantly different from that of FO. This result suggests that GSL has the same protective effect as FO against MNU-damaged retinal ERGs. 

Secondly, our results show that GSL, like FO, protects the retinal microstructure and ultrastructure against MNU-induced damage. Damage to the rat retinal photoreceptor cell induced by MNU was alleviated significantly in the GSL- and FO-treated groups, as observed by light microscopy. Under electron microscopy, GSL and FO were seen to reverse the damage to photoreceptor segments and photoreceptor cell nuclei induced by MNU. 

The major fatty acid constituents of human retinal lipids are palmitic (PA, 16:0n), stearic (SA, 18:0n), OA (18:1n-9), arachidonic (ARA, 20:4n-6), and DHA (22:6n-3) [18]. It does not differ significantly in fatty acid composition from the vitamin A ester mixture (containing C_12_ to C_18_ fatty acids) synthesized by the retinal tissue of other vertebrates [19]. Retinal neurons are highly enriched in lipids containing PUFAs, essential fatty acids that must be obtained from the diet. Diet supplementation with GSL, FO, or DHA in animals has the disadvantages of difficulty of accurately measuring daily intake and susceptibility to oxidation. Therefore, we used intragastric administration of GSL and FO in the present study to avoid these disadvantages.

FO, containing high levels of DHA and eicosapentaenoic acid (EPA, 20:5n-3), is known to influence membrane fatty acid composition and to modulate the formation of eicosanoids in several tissues [20, 21]. There is a generally accepted pathway for metabolism of EPA to DHA [22]. The dietary intake of n-3 fatly acids has not only important physiological consequences but also a therapeutic effect on retinal degeneration in vertebrate retina. Via binding to interphotoreceptor retinoid-binding protein, DHA release the 11-cis-retinal and is taken up by the photoreceptor cell [23]. DHA seems to act as a trophic molecule in photoreceptor survival both in vivo and in vitro [24]. Some studies have found lower DHA content in an RP animal model and RP patients [25, 26]. DHA supplementation of the diet of SD rats with retinal photoreceptor apoptosis induced by MNU both delayed the onset of photoreceptor cell apoptosis and suppressed the progression of retinopathy [24, 27]. In rats with retinal damage induce by kainic acid, increasing DHA levels in retinal and serum increased the b-wave amplitude of ERG and apparently also increasing the amount of ganglion and the thickness of the inner nuclear cell layer [28]. The FO we used in this research contained 70% n-3 PUFAs, of which DHA accounted 54% and EPA for 7% (data provided by Croda, UK).

Furthermore, FO contains both linoleic acid (LA, 18:2n-6) and OA as well [29]. Retinal photoreceptors matrix is high in OA [30], which was found to bind more tightly to interphotoreceptor retinoid-binding protein than did DHA. But LA serves as a substrate, and once obtained from the diet, it can be further metabolized into ARA and EPA [31]. LA and OA may both play a role in the FO inhibition of MNU-induced retinal damage. 

Potential therapeutic interventions using Ganoderma lucidum spores have attracted extensive interest as studies have suggested that extracts of these spores mediate neuronal neuroprotection [32]. The major bioactive components in Ganoderma lucidum spores are triterpenoids, polysaccharides, fatty acids, and steroids, similar to the Ganoderma itself [8], but their bioactivity may be much higher than that of the whole mushroom [33, 34]. Moreover, GSL extracted from sporoderm-broken spores by supercritical CO_2_ shows even higher bioactivity than the whole spores [34]. Sixteen fatty acids (C_14_ to C_24_) were identified in GSL provided by Holistol International Ltd., of which PA accounted for 14.55%, and SA 5.03%, LA 12.20%, OA 61.17%, linolenic acid 0.036%, and so forth (detected by China National Analytical Center, Guangzhou).

The neuroprotective mechanisms of FO are likely multifactorial, including prevention neuronal accumulation of calcium, depletion activates caspase, inhibition of glutaminergic transport, and neurotransmission involved excitotoxicity [35, 36]. The ability of n-3 PUFA to reduce oxidative stress may be an important component of its overall neuroprotective actions [5]. Lines of evidence also suggest that n-3 PUFA can affect the expression of many genes in the central nervous system, number of which represent transcription factors [37]. Furthermore, the protective effects of n-3 PUFA may attribute to alteration of nerve cell lipid composition and metabolism [38, 39]. In recent years, a number of studies have been published illustrating the potential protective effects of n-3 supplementation in ocular diseases such as age-related maculopathy [40]. And DHA can suppress photoreceptor cell apoptosis in the rat with MNU-induced retinal degeneration [24, 27]. DHA can dramatically increase differentiation in Crx-positive photoreceptor cells, enhancing opsin expression, apical differentiation, and axonal outgrowth [41]. 

Similarly, GSL riches in PUFAs, and companying by other bioactive compositions exhibits the neuroprotective effects via antioxidative stress [9], increasing neuronal differentiation [10], and preventing toxicity effects on neurons [11]. Cheung and colleagues [10] reported that Ganoderma extract induced the neuronal differentiation of PC12 cells from apoptosis, and the effects may be mediated via the Ras/Erk and cAMP-response element-binding protein signaling pathways. Moreover, oxidative stress has been linked with the pathogenesis of retinal degeneration. Ganoderma Lucidum peptide is the major antioxidant component of Ganoderma Lucidum and may be involved in the inhibition of lipid peroxidation through its antioxidant, metal chelating, and free radical-scavenging activities [42]. The polysaccharides, also major components of Ganoderma Lucidum, can effectively reduce oxidative injury and inhibit apoptosis by increasing antioxidant enzyme activities and modifying Bcl-2 expression and Bax/Bcl-2 ratio [43].

Our previous study suggested that GSL may regulate the expressions of Bax, Bcl-xl, and caspase-3, inhibiting MNU-induced rat photoreceptor cell apoptosis and protecting retinal function in a dose-dependent manner [12]. In the present paper, we compared the protective effect of GSL and FO on lesion of rat retinal visual function, photoreceptor cell damage, and loss induced by MNU using electrophysiology and morphology methods. Our data indicate that GSL is similar to FO in alleviating the extent of rat retinal photoreceptor cell damage induced by MNU.

Either GSL or FO contains a great number of PUFAs. There are two major classes of PUFAs: n-3 (e.g., DHA, EPA, and *α*-linolenic acid) and n-6 (e.g., LA, ARA, and *γ*-linolenic acid). Both n-3 and n-6 PUFAs play important roles in neuronal growth, development of synaptic processing of neuronal cell interaction, and expression of genes regulating cell differentiation and growth [44]. Schaebelen and co-workers [45] demonstrated that a 6-month supplementation with a combination of n-3 (EPA and DHA) and n-6 PUFAs (*γ*-linolenic acid) was more effective than single supplementation, and the EPA+DHA+*γ*-linolenic acid dietary combination prevented retinal damage induced by the elevation of intraocular pressure in rats. GSL and FO obtained from Ganoderma lucidum and sea fish, respectively, are the nature mixture of n-3 and n-6 PUFAs. So, these lines of evidence support our results that GSL and FO have the same roles of inhibition of retinal damage induced by MNU. 

Although GSL protects retina similar to FO, it has certain advantages over FO. First, GSL can be obtained on a commercial scale from Ganoderma lucidum farming, making it a potentially cheap and plentiful substance. FO only can be abstracted from sea fish. Increasing production and consumption of GSL to substitute FO can avoid depending on sea fish and relieve the burden of overfishing. Second, GSL is well tolerated and has fewer side effects than FO. It has the potential to be useful to those who cannot take FO. This additional benefit of GSL in combating MNU-induced rat photoreceptor cell apoptosis and protecting retinal function indicates the need for further research into other potential therapeutic applications.

## Figures and Tables

**Table 1:** The grading criteria for outer nuclear layer (ONL) damage induced by MNU.

Grade	ONL damage	Score
0	Normal appearance	0

1	Distorted nuclear orientation ≤1/2 normal ONL thickness but no apparent reduction in ONL thickness	1

2	Distorted nuclear orientation >1/2 normal ONL thickness, or cell loss ≤1/4 normal ONL thickness and ≤1/6 complete length of retinal section	2

3	Cell loss of 1/4–1/3 normal ONL thickness and >1/6 complete length of retinal section	3

4	Cell loss of 1/3–1/2 normal ONL thickness and >1/6 complete length of retinal section	4

5	Cell loss ≥1/2 normal ONL thickness and >1/6 complete length of retinal section	5
